# Supplementary material for: RNA G-quadruplex in TMPRSS2 reduces SARS-CoV-2 infection
Source: Nat Commun. 2022 Mar 17;13:1444. doi: 10.1038/s41467-022-29135-5 (PMC8931161; doi:10.1038/s41467-022-29135-5)
Supplement: Supplementary file 1 — Supplementary Information [file 41467_2022_29135_MOESM1_ESM.pdf]

Supplementary materials

Supplementary Fig. 1 Characterization of RG4 in SARS-CoV-2 genome and host factors.

Supplementary Fig. 2 RG4 inhibits human TMPRSS2 expression.

Supplementary Fig. 3 RG4 stabilizers inhibit SARS-CoV-2 pseudoviruses entry.

Supplementary Fig. 4 RG4 inhibits murine TMPRSS2 protein expression.

Supplementary Fig. 5 RG4 inhibits SARS-CoV-2 pseudoviruses entry *in vivo*.

Supplementary Fig. 6 TMPRSS2 is induced in lungs of patients with COVID-19.

Supplementary Fig. 7 The website screenshot of predicted RG4 regions in Axl (left) and Furin (right) from the QGRS-Mapper.

Supplementary Table 1 Putative RG4s in SARS-CoV-2 genome.

Supplementary Table 2 Putative RG4s in human *Tmprss2* and *Ace2*.

Supplementary Table 3 The melting temperature (Tm, ° C) of PQS-675-WT and PQS-675-Mut RNA under KCl, LiCl, or PDS conditions.

Supplementary Table 4 Putative RG4s in mouse *Tmprss2*.

Supplementary Table 5 Sequences of oligomers used in this study.

Supplementary Table 6 Primers used in this study.

Supplementary Fig. 1

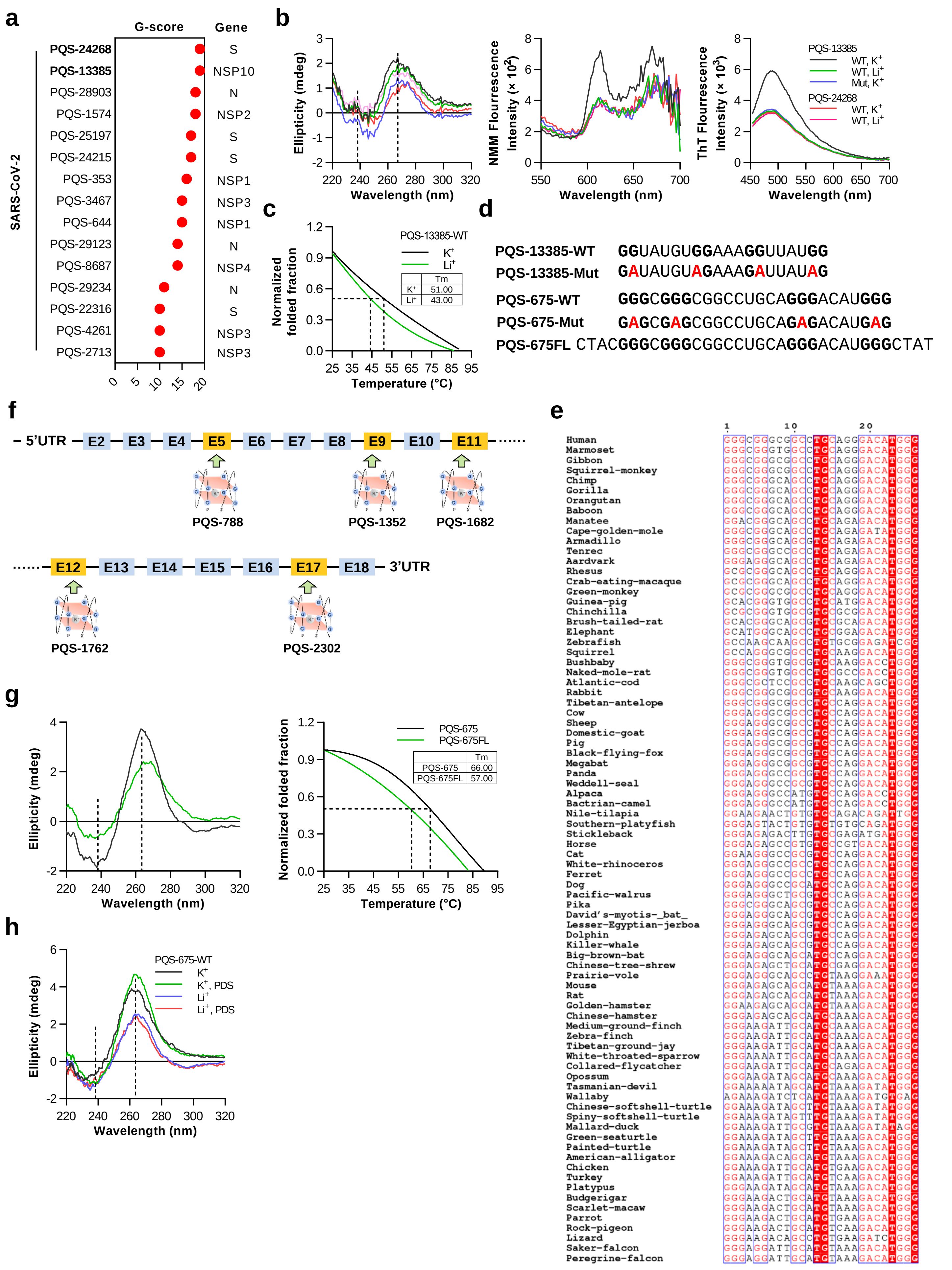

Supplementary Fig. 1 Characterization of RG4 in SARS-CoV-2 genome and host factors.

a RG4 potential of SARS-CoV-2 genome was predicted by QGRS-Mapper.

b CD spectrum (left panel), NMM (middle panel) and ThT (right panel) fluorescence emission spectra of PQS-13385-WT, PQS-13385-Mut and PQS-24268-WT RNA under KCl or LiCl conditions.

c CD melting measurements of PQS-13385-WT RNA under KCl or LiCl conditions.

d The WT and mutant RG4 sequence of PQS-13385, PQS-675 and PQS-675FL mRNA used for RG4 characterization.

e Conservation of PQS-675 sequence.

f Five top PQS sites in *Ace2* mRNA.

g CD spectra (left panel) and CD melting measurements (right panel) of PQS-675 and PQS-675FL RNA under KCl condition.

h CD spectrum of PQS-675-WT RNA under KCl, LiCl or PDS conditions.

Source data are provided as a source data file.

Supplementary Fig. 2

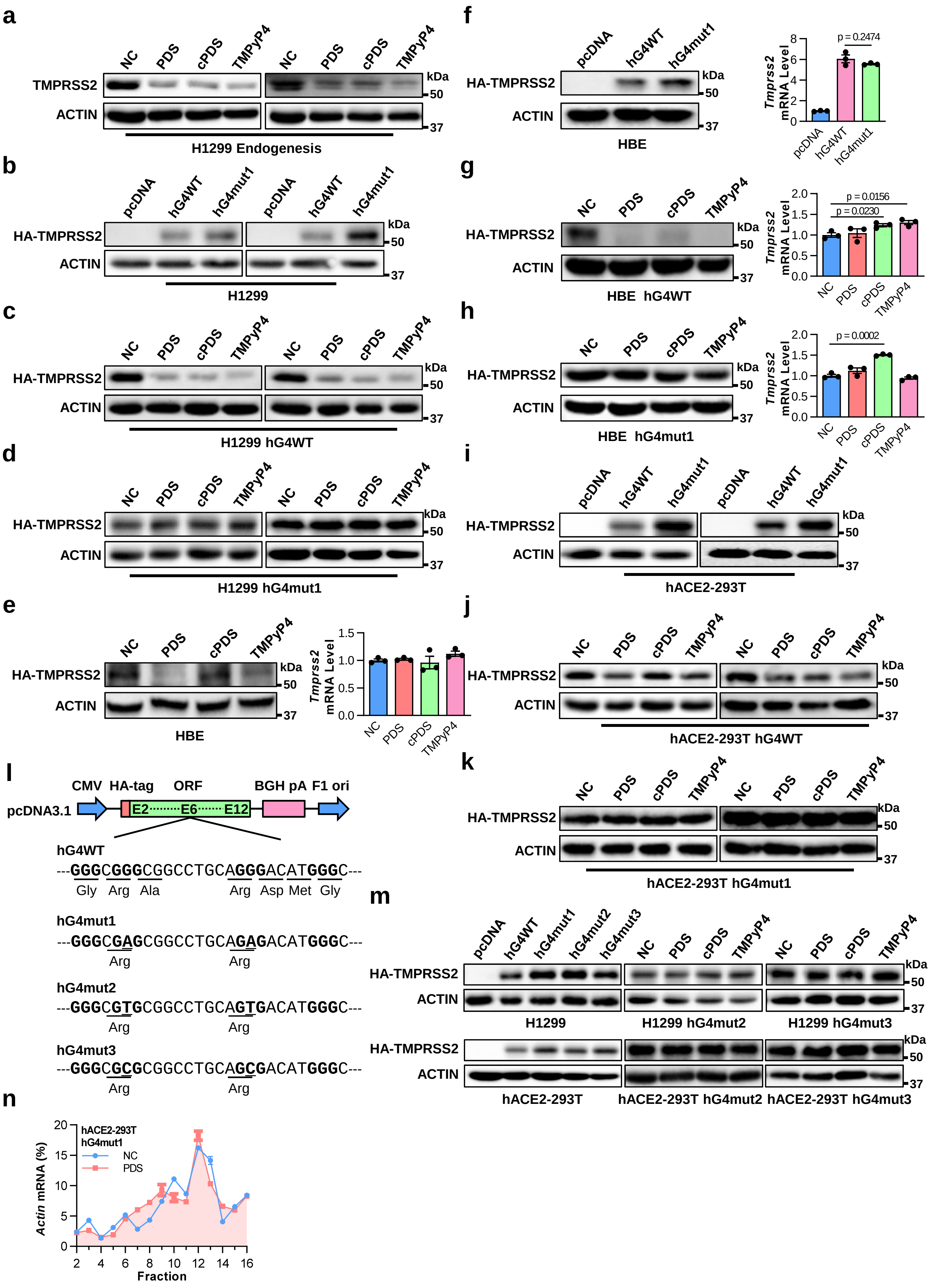

Supplementary Fig. 2 RG4 inhibits human TMPRSS2 expression.

a-d The replicate image of Western blot for Fig. 2a, c-e.

e Levels of endogenous TMPRSS2 protein (left panel), and mRNA (right panel) in HBE cells treated with PDS (10 μM), cPDS (10 μM), or TMPyP4 (10 μM).

f Levels of HA-TMPRSS2 protein (left panel), and mRNA (right panel) in HBE cells transfected with hG4WT or hG4mut1 plasmids.

g, h Levels of HA-TMPRSS2 protein (left panel), and mRNA (right panel) in HBE cells transfected with hG4WT (**g**) or hG4mut1 (**h**) plasmids, and stimulated with PDS (10 μM), cPDS (10 μM), or TMPyP4 (10 μM).

i-k The replicate image of Western blot for Fig. 2f-h.

l Schematic for different synonymous mutations of the TMPRSS2 expression plasmid.

m Levels of HA-TMPRSS2 protein in H1299 (top panel) and hACE2-293T (bottom panel) cells transfected with hG4WT, hG4mut2 and hG4mut3 plasmids, and stimulated with PDS (10 μM), cPDS (10 μM), or TMPyP4 (10 μM).

n Polysome shift analysis of endogenous *Actin* mRNA in hACE2-293T cells transfected with hG4mut1plasmids and treated with PDS (10 μM).

Data are shown as mean ± SEM, n = 3. Two-tailed Student's *t* test. Source data are provided as a source data file.

Supplementary Fig. 3

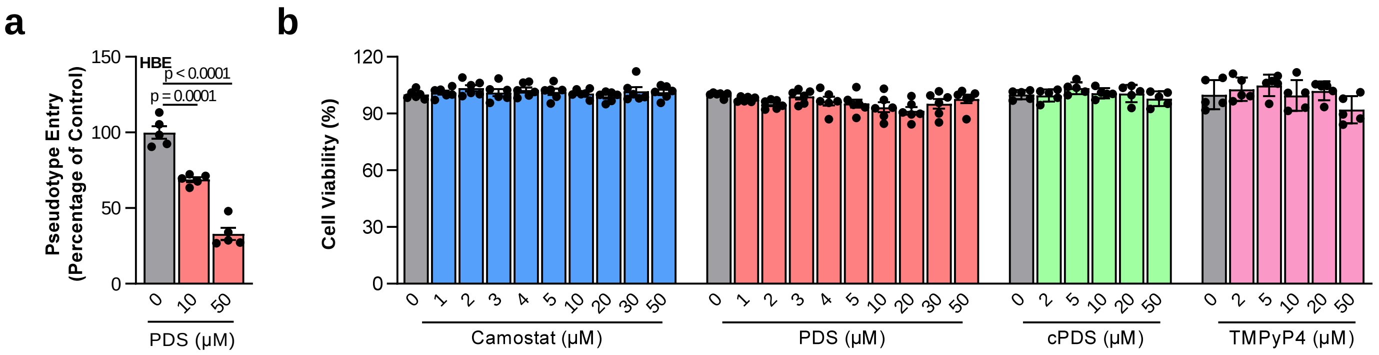

Supplementary Fig. 3 RG4 stabilizers inhibit SARS-CoV-2 pseudoviruses entry.

a SARS-CoV-2 pseudoviruses entry efficiency in HBE cells treated with PDS.

b Cytotoxicity of camostat mesylate, PDS, cPDS and TMPyP4 in H1299 cells was assessed by MTS assay.

Data are shown as mean ± SEM, n = 5. Two-tailed Student's *t* test. Source data are provided as a source data file.

Supplementary Fig. 4

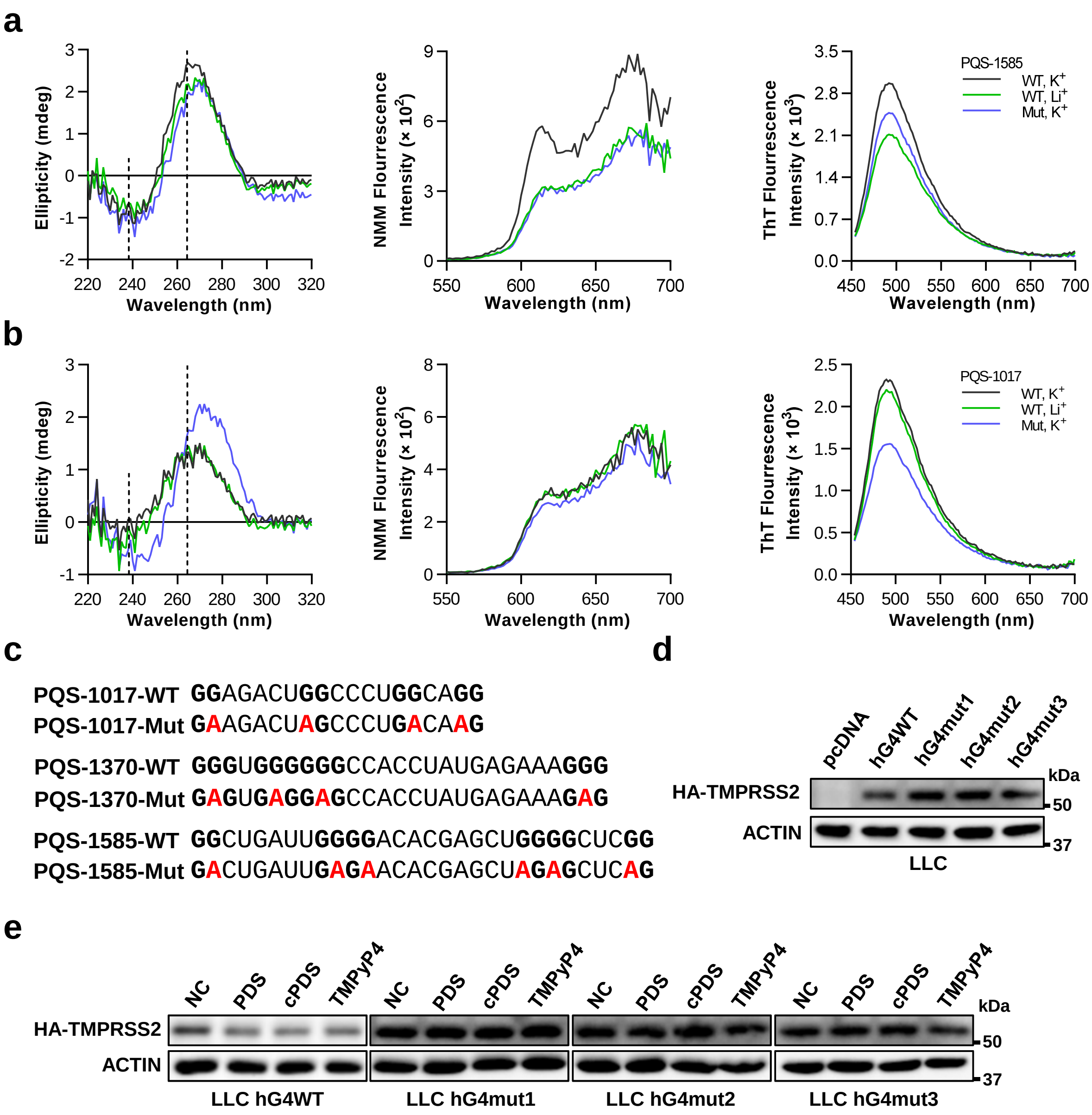

Supplementary Fig. 4 RG4 inhibits murine TMPRSS2 protein expression.

a, b CD spectrum (left panel), NMM (middle panel) and ThT (right panel) fluorescence emission spectra of WT and RG4-Mut RNAs of PQS-1585 (**a**) and PQS-1017 (**b**) under KCl or LiCl conditions.

c The WT and mutant RG4 sequence of PQS-1370, PQS-1585 and PQS-1017 in mouse *Tmprss2* mRNA used for RG4 characterization.

d Levels of human HA-TMPRSS2 protein in LLC cells transfected with hG4WT, hG4mut1, hG4mut2 and hG4mut3 plasmids.

e Levels of human HA-TMPRSS2 protein in LLC cells transfected with hG4WT, hG4mut1, hG4mut2 and hG4mut3 plasmids, and treated with PDS (10 μM), cPDS (10 μM), or TMPyP4 (10 μM).

All data represented at least three independent experiments. Source data are provided as a source data file.

Supplementary Fig. 5

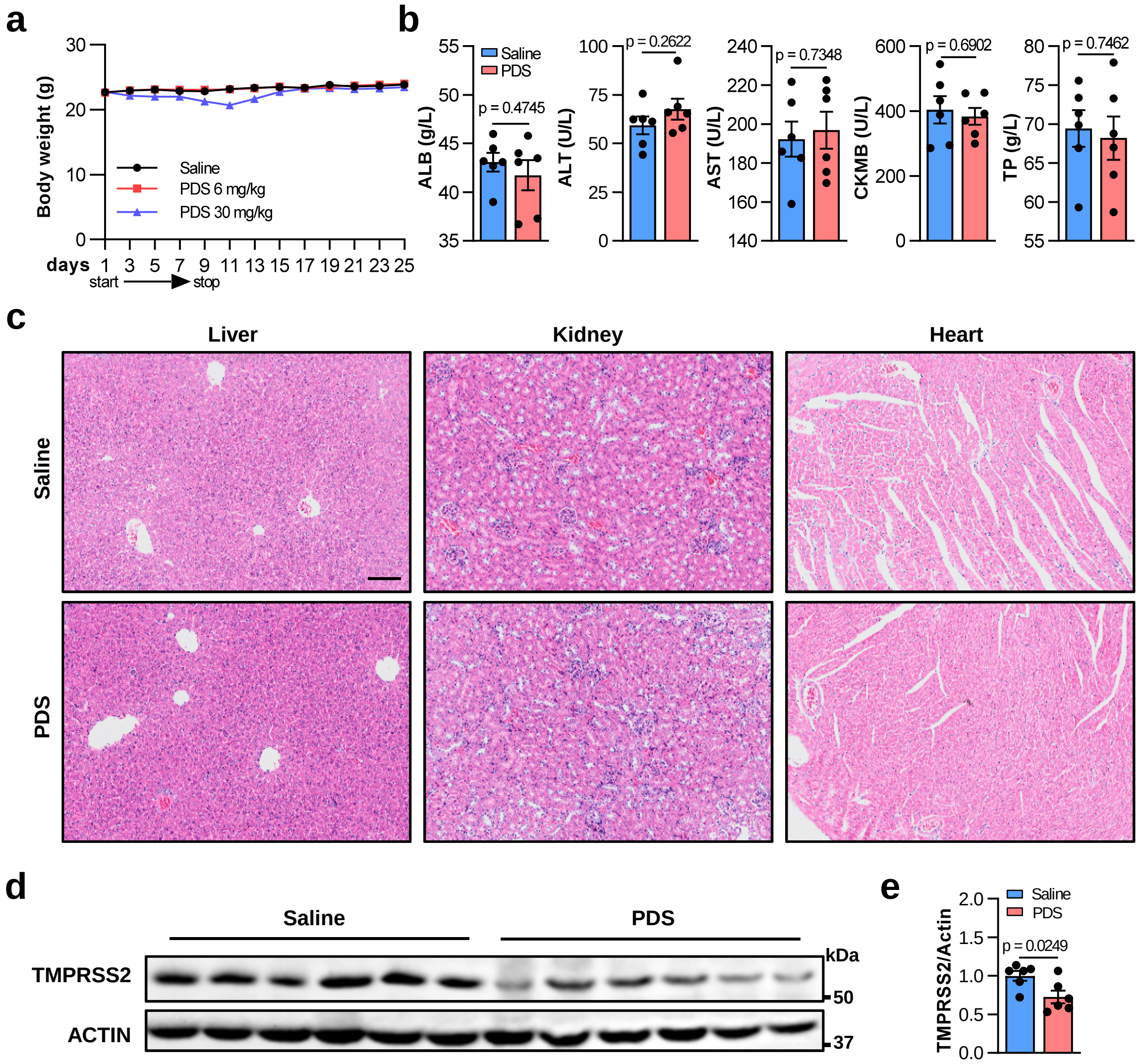

Supplementary Fig. 5 RG4 inhibits SARS-CoV-2 pseudoviruses entry *in vivo*.

a 6- to 8-week-old C57BL/6J mice were infused with 6 mg/kg, 30 mg/kg body weight of PDS, or saline via caudal vein daily for 9 days. Mouse body weights were recorded every two days and shown (n = 6).

b Clinical chemistry and hematological parameters (ALB, ALT, AST, CKMB and TP) from the peripheral blood of the VSV-SARS-2-S-luc-infected mice.

c Representative H&E staining of paraffin liver, kidney and heart sections. Scale bars: 100 μm.

d, e Levels of TMPRSS2 protein in livers of VSV-SARS-2-S-luc-infected mice. ImageJ quantification of the TMPRSS2/ACTIN ratio in (**d**) is shown (**e**).

Data are shown as mean ± SEM, n = 6. Two-tailed Student's *t* test. Source data are provided as a source data file.

Supplementary Fig. 6

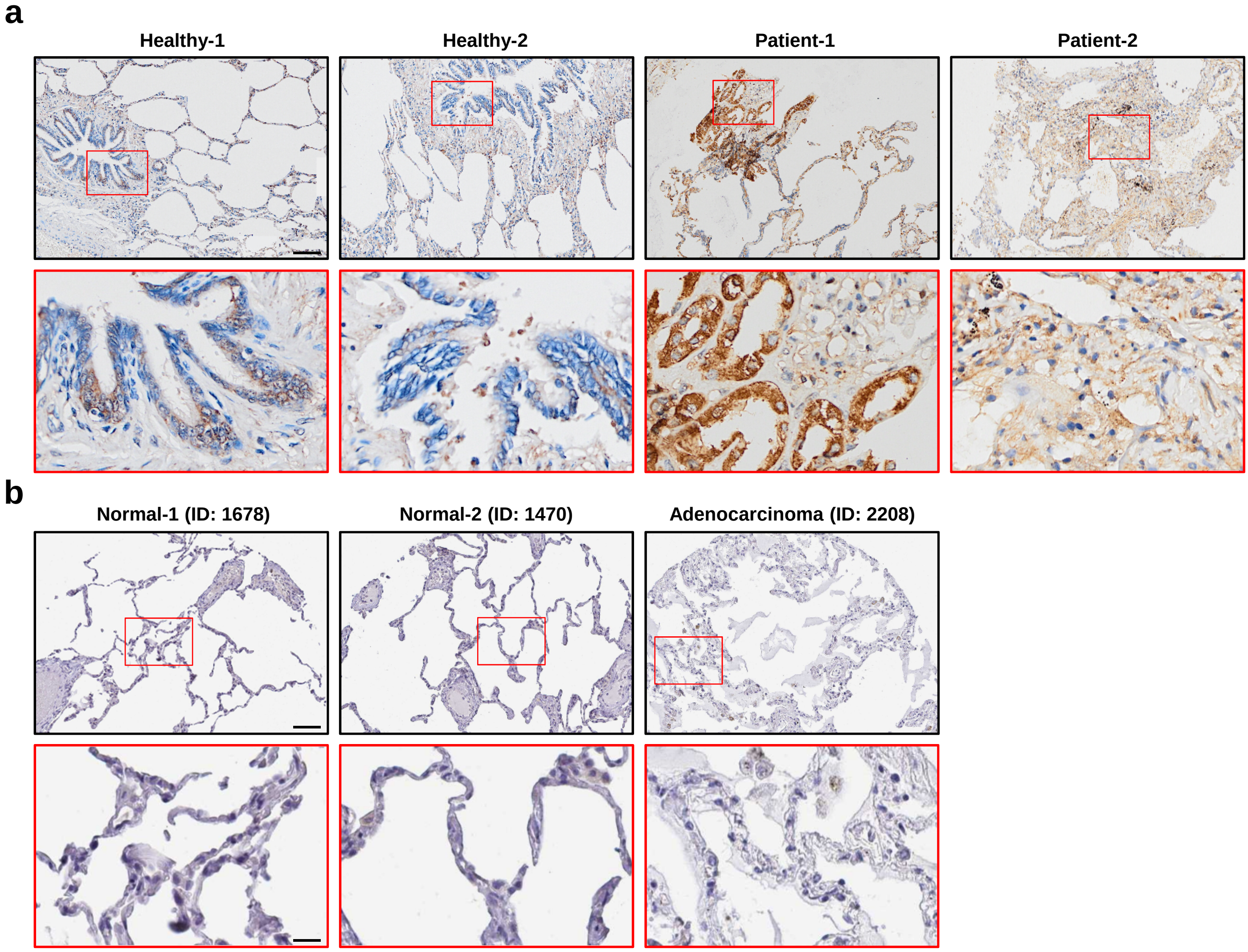

Supplementary Fig. 6 TMPRSS2 is induced in lungs of patients with COVID-19.

a IHC staining analysis for TMPRSS2 in human lung tissue. Representative samples from two healthy people with no underlying chronic airway disease, and two patients with COVID-19.

b IHC images of TMPRSS2 in human lung tissue from three SARS-CoV-2 uninfected people detected in the HPA database (https://www.proteinatlas.org/).

Black outlines: low magnification of conducting airways with airway epithelium; scale bars: 100 μm. Red outlines: high magnification of conducting airway epithelium that are defined in the low magnification images by red squares; scale bars: 20 μm.

All data represented at least three independent experiments.

Supplementary Fig. 7

Export to Excel

Data View

Search Parameters: QGRS Max Length: 30 | Min G-Group Size: 2 | Loop size: from 0 to 36 | Loop search string:

QGRS sequences found (overlaps not included)

Position	Length	QGRS	G-Score
21	15	GGGGGGAGGGCCGGG	40
94	28	GGCACCCATGGCGTGGCGGTGCCCCAGG	20
125	21	GGCAGGGTCCCGCTGGCCTGG	17
166	29	GGCGTGCATGGCCCCCAGGGGCACGCAGG	21
236	30	GGTGCCCGGGGACTCACGGGCACCCTTCGG	20
298	21	GGTACATTGGCTTCGGGATGG	18
370	26	GGATGAACAGGATGACTGGATAGTGG	19
491	20	GGCTATGTTGGGCTGGAGGG	16
526	26	GGAGGAGCCCGAAGACAGGACTGTGG	10
640	21	GGCCACGGCTCCAGGTCACGG	20
817	27	GGAGCTGGAGGTGGCTTGGACTCCAGG	20
903	19	GGATGGGCATCCAGGCGGG	17
1059	30	GGACCCACTGGCTTCCTGTGGAGACGCCGG	20
1131	29	GGAATGGGAGCCAGGCCTTCGTGCATTGG	13
1197	27	GGTACCGGCTGGCGTATCAAGGCCAGG	16
1243	23	GGACATAGGGCTAAGGCAAGAGG	21
1273	18	GGAGCTGCAGGGGGACGG	15
1337	15	GGGGATGGACCCTGG	16
1369	18	GGAGGCCTGGCGCCCAGG	16
1443	25	GGCCCTGGTGGTATGTACTGCTAGG	10
1567	25	GGAAAGAGGTGAACTGGTAGTCAGG	20
1669	26	GGAGAAGCTGCGGGATGTGATGGTGG	13
1698	30	GGCACAAGGTGGCCCTGGGGAAGACTCTGG	19
1733	26	GGAGAGTTTGGAGCTGTGATGGAAGG	14
2113	16	GGACCTGGCGGCCAGG	18
2298	23	GGTCCTTCGGGGTGACAATGTGG	12
2451	29	GGTGCTGGGAGCTAAATCCCCAGGACCGG	11
2572	15	GGATGAGGGTGGAGG	18
2766	13	GGCAGGAGGATGG	20
2993	12	GGAAGGGGTTGG	19
3384	25	GGTTTCACCATGTTGGCCAGGCTGG	11
3706	19	GGAGGTTCTAAGGCCTAGG	16
4498	27	GGATGCCTCCTTTCCCGCAGGGGATGG	4
4593	29	GGACTCTGGTGCCTCCAGAGGGGCTCAGG	16

Export to Excel

Data View

Search Parameters: QGRS Max Length: 30 | Min G-Group Size: 2 | Loop size: from 0 to 36 | Loop search string:

QGRS sequences found (overlaps not included)

Position	Length	QGRS	G-Score
11	29	GGAAGGGCCCCGCCCTGTGAAGGGATAGG	10
67	24	GGCGGGGAAGCAGCAGCGGCCAGG	14
101	30	GGTGCTCTGGAGCTGGATGGTGAAGGTCGG	19
198	29	GGCAGTGAGCAGGCACCTGGGAGCCGAGG	18
238	24	GGCCAAGGAGACGGGCGCTCCAGG	18
286	25	GGAGCTGAGGCCCTGGTTGCTATGG	18
401	16	GGAGGCCCAGCGGTGG	16
427	29	GGCACGGAAGCATGGGTTCCTCAACCTGG	13
467	29	GGGGACTATTACCACTTCTGGCATCGAGG	6
567	18	GGCTGGAACAGCAGGTGG	15
688	27	GGCGGCCTGGGCGCAGGGCTACACAGG	20
719	29	GGCATTGTGGTCTCCATTCTGGACGATGG	16
766	27	GGACTTGGCAGGCAATTATGATCCTGG	10
861	21	GGCACGGCACACGGTGTGCGG	19
889	30	GGCTGCGGTGGCCAACAACGGTGTCTGTGG	20
947	28	GGAGGGGTGCGCATGCTGGATGGCGAGG	14
1044	30	GGGGCCCCGAGGATGACGGCAAGACAGTGG	17
1113	28	GGGTTAGCCAGGGCCGAGGGGGGCTGGG	39
1155	24	GGGCCTCGGGGAACGGGGGCCGGG	42
1452	24	GGCGGGACATGCAACACCTGGTGG	9
1516	22	GGCCACCAATGGTGTGGGCCGG	16
1556	23	GGCTACGGGCTTTTGGACGCAGG	21
1588	29	GGCCCTGGCCCAGAATTGGACCACAGTGG	16
1667	21	GGGAAACGGCTCGAGGTGCGG	19
1731	23	GGCTGGAGCACGCTCAGGCGCGG	13
1899	27	GGGATGAGGATCCCTCTGGCGAGTGGG	18
2065	24	GGCCTGTGTGGTGTGCGAGGAAGG	16
2371	11	GGAGGTGGAGG	21
2385	20	GGCAACGGCTGCGGGCAGGG	21
2428	12	GGTGGTGGCCGG	20
2518	30	GGGGGTGAAGGTGTACACCATGGACCGTGG	16
2565	27	GGCTGCCCCCTGAAGCCTGGCAGGAGG	6
2614	22	GGACGAGGGCCGGGGCGAGAGG	20
2768	18	GGGAGGCAAGAGGGGTGG	18
2829	30	GGTGGGCCCAGGACCAGCTGGGGCGTGGGG	20
3054	22	GGCAGTCGGGGGCTGGCCTAGG	20
3087	22	GGAGGAGGCCACCTCTCCAAGG	10
3175	29	GGGACCAAGGCAAGGCAGGTGCCTCCAGG	19
3276	25	GGCCACCAGGCTGGCGCAGCCAAGG	14
3441	20	GGGTGGGTGGTGGGGAGGGG	39
3515	22	GGATCTCAGGGGCTGTTTGAGG	13
3658	27	GGCTGCCCTGGCCCTGAGGTGTGGGGG	20
3753	27	GGGCTCAAGGAAAGGGGGTCCCAGTGG	20
3782	30	GGGGCAGGCTGACATCTGTGTTTCAAGTGG	1
3825	20	GGGGGTTCATAGGTCACTGG	16
3861	13	GGTGGGCAGGTGG	20
4103	27	GGCTGGTTTTGTAAGATGCTGGGTTGG	9

Supplementary Fig. 7 The website screenshot of predicted RG4 regions in *Axl* (left) and *Furin* (right) from the QGRS-Mapper (https://bioinformatics.ramapo.edu/QGRS/index.php).

Supplementary Table 1. Putative RG4s in SARS-CoV-2 genome.

Site	Gene	G-score	Putative RG4 sequence
353	NSP1	16	**GG**CUUU**GG**AGACUCCGU**GG**A**GG**A**GG**
644	NSP1	15	**GG**UAAUAAA**GG**AGCU**GG**U**GG**
1574	NSP2	18	**GG**UGUUGUU**GG**AGAA**GG**UUCCGAA**GG**
2713	NSP3	10	**GG**C**GG**UGCACCAACAAA**GG**UUACUUUU**GG**
3467	NSP3	15	**GG**A**GG**A**GG**UGUUGCA**GG**
4261	NSP3	10	**GGG**UUUAAAU**GG**UUACACUGUAGA**GG**A**GG**
8687	NSP4	14	**GG**AUACAA**GG**CUAUUGAU**GG**U**GG**
13385	NSP10	19	**GG**UAUGU**GG**AAA**GG**UUAU**GG**
22316	S	10	**GG**UGAUUCUUCUUCAGGUU**GG**ACAGCU**GG**
24215	S	17	**GG**UU**GG**ACCUUU**GG**UGCA**GG**
24268	S	19	**GG**CUUAUA**GG**UUUAAU**GG**UAUU**GG**
25197	S	17	**GG**CCAU**GG**UACAUUU**GG**CUA**GG**
28903	N	18	**GG**CU**GG**CAAU**GG**C**GG**
29123	N	14	**GG**AAAUUUU**GGGG**ACCA**GG**
29234	N	11	**GG**CAU**GG**AAGUCACACCUUC**GGG**AACGU**GG**

Note: G-tracts in the putative RG4 region are indicated in **bold**.

Supplementary Table 2. Putative RG4s in human *Tmprss2* and *Ace2*.

Site	Position	G-score	Conservation	Putative RG4 sequence
Tmprss2				
21	5'UTR	21	-0.9102	**GG**A**GG**C**GG**A**GG**
33	5'UTR	21	-0.8050	**GG**A**GG**C**GG**A**GG**
675	ORF	34	1.8725	**GGG**C**GGG**CGGCCUGCA**GGG**ACAU**GGG**
1281	ORF	28	2.9465	**GGG**U**GGGGGG**CCACCGA**GG**AGAAA**GGG**
2303	3'UTR	38	-1.0705	**GGG**U**GGGG**CUCCU**GGG**A**GGG**
Ace2				
788	ORF	19	4.8796	**GG**CUCU**GGG**CUU**GGG**AAAGCU**GG**
1352	ORF	18	4.3577	**GGG**ACCU**GGGG**AA**GGG**CGACUUCA**GG**
1682	ORF	20	5.3713	**GG**A**GG**U**GG**AU**GG**
1762	ORF	20	4.1023	**GGGG**U**GG**U**GG**
2302	ORF	20	0.3421	**GGGG**A**GG**A**GG**

Note: G-tracts in the putative RG4 region are indicated in **bold**.

Supplementary Table 3. The melting temperature (Tm, °C) of PQS-675-WT and PQS-675-Mut RNA under KCl, LiCl, or PDS conditions.

Group	Tm (CD-melting)	Tm (FRET-melting)
**PQS-675-WT**		
K^+	70.00	74.00
K^+, PDS	74.00	77.48
Li^+	64.00	63.27
Li^+, PDS	64.00	65.71
**PQS-675-Mut**		
K^+	N/A	38.33
K^+, PDS	N/A	38.03

Supplementary Table 4. Putative RG4s in mouse *Tmprss2*.

Site	Position	G-score	Putative RG4 sequence
1017	ORF	18	**GG**AGACU**GG**CCCU**GG**CA**GG**
1141	ORF	16	**GG**UACU**GG**AC**GG**CAUUUGC**GG**
1371	ORF	28	**GGG**U**GGGGGG**CCACCUAUGAGAAA**GGG**
1539	ORF	16	**GG**AGACAGU**GG**AG**GG**CCGCU**GG**
1585	ORF	17	**GG**CUGAUU**GGGG**ACACGAGCU**GGGG**CUC**GG**

Note: G-tracts in the putative RG4 region are indicated in **bold**.

Supplementary Table 5. Sequences of oligomers used in this study.

Name	Sequence (from 5'-3')
**SARS-CoV-2 genome**	
PQS-13385-WT	GGUAUGUGGAAAGGUUAUGG
PQS-24268-WT	GGCUUAUAGGUUUAAUGGUAUUGG
PQS-13385-Mut	GAUAUGUAGAAAGAUUAUAG
**Human *Tmprss2***	
PQS-675-WT	GGGCGGGCGGCCUGCAGGGACAUGGG
PQS-675-Mut	GAGCGAGCGGCCUGCAGAGACAUGAG
PQS-675FL-WT	CTACGGGCGGGCGGCCUGCAGGGACAUGGGCTAT
PQS-675FL-Mut	CTACGAGCGAGCGGCCUGCAGAGACAUGAGCTAT
PQS-675-WT-FAM	FAM-GGGCGGGCGGCCUGCAGGGACAUGGG
PQS-675-Mut-FAM	FAM-GAGCGAGCGGCCUGCAGAGACAUGAG
PQS-675-WT-FRET	FAM-GGGCGGGCGGCCUGCAGGGACAUGGG-TAMRA
PQS-675-Mut-FRET	FAM-GAGCGAGCGGCCUGCAGAGACAUGAG-TAMRA
PQS-675-WT-biotin	Biotin-GGGCGGGCGGCCUGCAGGGACAUGGG
PQS-675-Mut-biotin	Biotin-GAGCGAGCGGCCUGCAGAGACAUGAG
PQS-1281-WT	GGGUGGGGGGCCACCGAGGAGAAAGGG
ASO	TCCCTGCAGGCCGCCCGCCC
**Mouse *Tmprss2***	
PQS-1370-WT	GGGUGGGGGGCCACCUAUGAGAAAGGG
PQS-1585-WT	GGCUGAUUGGGGACACGAGCUGGGGCUCGG
PQS-1017-WT	GGAGACUGGCCCUGGCAGG
PQS-1370-Mut	GAGUGAGGAGCCACCUAUGAGAAAGAG
PQS-1585-Mut	GACUGAUUGAGAACACGAGCUAGAGCUCAG
PQS-1017-Mut	GAAGACUAGCCCUGACAAG

Supplementary Table 6. Primers used in this study.

Genes	Primers (from 5’-3’)
Human Tmprss2	F: GCAGTGGTTTCTTTACGCTGT
	R: CCGCAAATGCCGTCCAATG
Mouse Tmprss2	F: ATGCTCCGAGGATTACAACGC
	R: CGAGGGCTAAACACAGCGATT
Human Actin	F: GTTGCTATCCAGGCTGTGCT
	R: AGGGCATACCCCTCGTAGAT
Mouse Actin	F: TAGGCACCAGGGTGTGATGG
	R: CATGGCTGGGGTGTTGAAGG
Renilia	F: GCTGGACTCCTTCATCAACTACTA
	R: GACTTACCCATTCCGATCAGATCA
